# Localized DNA tetrahedrons assisted catalytic hairpin assembly for the rapid and sensitive profiling of small extracellular vesicle-associated microRNAs

**DOI:** 10.1186/s12951-022-01700-6

**Published:** 2022-12-01

**Authors:** Ye Zhang, Wenbin Li, Tingting Ji, Shihua Luo, Jiuxiang Qiu, Bo Situ, Bo Li, Xiaohe Zhang, Tiange Zhang, Wen Wang, Yunju Xiao, Lei Zheng, Xiaohui Yan

**Affiliations:** 1 Laboratory Medicine Center, Department of Laboratory Medicine, Nanfang Hospital, Southern Medical University, Guangzhou, 510515 Guangdong China; 2 Guangdong Engineering and Technology Research Center for Rapid Diagnostic Biosensors, Nanfang Hospital, Southern Medical University, Guangzhou, 510515 China; 3 Department of Clinical Laboratory, Shunde Hospital, Southern Medical University (The First People’s Hospital of Shunde), Foshan, 528300 Guangdong China; 4 Department of Laboratory Medicine, Guangzhou Eighth People’s Hospital, Guangzhou Medical University, Guangzhou, 510515 China; 5 Center for Clinical Laboratory Diagnosis and Research, The Affiliated Hospital of Youjiang Medical University for Nationalities, Baise, 533000 Guangxi People’s Republic of China; 6 Division of Laboratory Medicine, Guangdong Provincial People’s Hospital, Guangdong Academy of Medical Sciences, Guangzhou, 510000 China

**Keywords:** DNA tetrahedron, Catalyzed hairpin assembly, sEV-miRNAs, Early cancer diagnostics

## Abstract

The profiling of small extracellular vesicle-associated microRNAs (sEV-miRNAs) plays a vital role in cancer diagnosis and monitoring. However, detecting sEV-miRNAs with low expression in clinical samples remains challenging. Herein, we propose a novel electrochemical biosensor using localized DNA tetrahedron-assisted catalytic hairpin assembly (LDT-CHA) for sEV-miRNA determination. The LDT-CHA contained localized DNA tetrahedrons with CHA substrates, leveraging an efficient localized reaction to enable sensitive and rapid sEV-miRNA measurement. Based on the LDT-CHA, the proposed platform can quantitatively detect sEV-miRNA down to 25 aM in 30 min with outstanding specificity. For accurate diagnosis of gastric cancer patients, a combination of LDT-CHA and a panel of four sEV-miRNAs (sEV-miR-1246, sEV-miR-21, sEV-miR-183-5P, and sEV-miR-142-5P) was employed in a gastric cancer cohort. Compared with diagnosis with single sEV-miRNA, the proposed platform demonstrated a higher accuracy of 88.3% for early gastric tumor diagnoses with higher efficiency (AUC: 0.883) and great potential for treatment monitoring. Thus, this study provides a promising method for the bioanalysis and determination of the clinical applications of LDT-CHA.

**Graphical Abstract:**

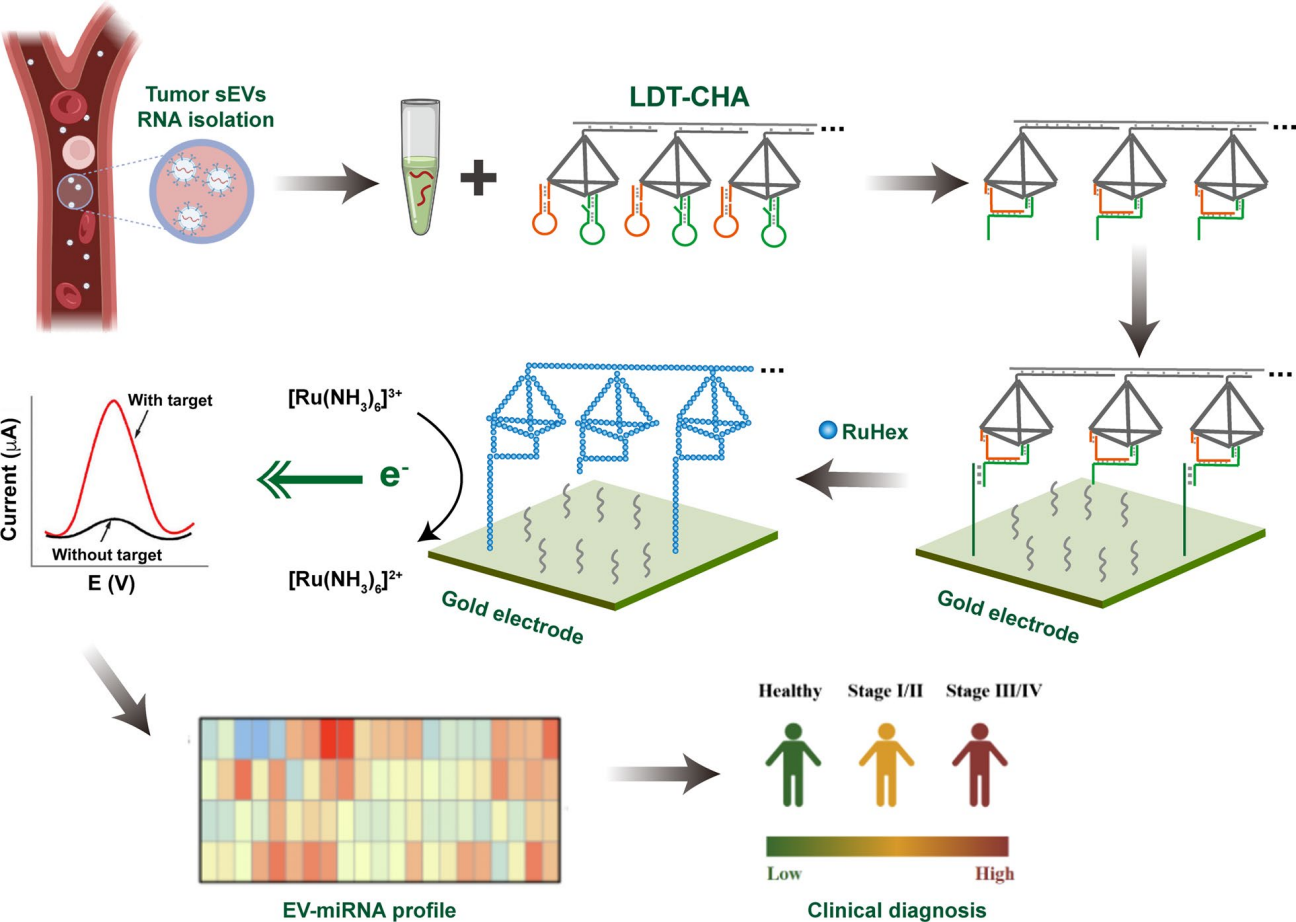

**Supplementary Information:**

The online version contains supplementary material available at 10.1186/s12951-022-01700-6.

## Introduction

Small extracellular vesicles (sEVs) with sizes ranging from 30 to 200 nm are lipid bilayer-packaged membrane vesicles shed by almost all cell types [1]. They can transmit valuable biological information to recipient cells by transferring intravesicular biomolecules from their parental cells [2]. MicroRNAs (miRNAs) are a class of noncoding RNAs that can be largely enclosed in sEVs [3]. They bind to target mRNAs to regulate gene expression, which participates in the occurrence and development of many diseases [4–6]. Compared to free miRNAs, EV-miRNAs can be more stably detected in circulation owing to their protection by sEV lipid membranes, which makes them potential biomarkers for cancer diagnosis [7, 8].

At present, the conventional method for sensitive sEV-miRNA determination is reverse transcription quantitative polymerase chain reaction (RT–qPCR) [9]. However, the clinical application of RT–qPCR is greatly impeded due to the tediousness of operation, specialized experimental equipment required, and high false positive rate [10, 11]. In this regard, multiple methods have been proposed for sEV-miRNA analysis, such as the use of nanomaterial-based technologies [12–14], enzyme-assisted strategies [15–17], and spherical nucleic acid [18–20]. Despite their decent performances, these strategies are not rapid and simple enough for sEV-miRNAs measurement because of the tedious operation and harsh reaction conditions. Accordingly, metastable hairpin-driven amplification, including catalytic hairpin assembly (CHA) [21–23], hybridization chain reaction (HCR) [24, 25], have been proposed due to its high specificity, low-cost, and easy to use. Compared with the assembled concatemer products of HCR, the dangled ssDNA products of CHA are easier to capture and generate an enhanced electrochemical signal [18, 26, 27]. However, the deficient sensitivity and inefficiency hindered the practical application of metastable hairpin-driven amplification.

In order to improve the sensitivity of metastable hairpin-driven amplification, multiple amplification were proposed [28–30]. Such as, Peng et al. proposed a multiple amplification strategy by integrating CRISPR-Cas12a and CHA for miRNAs detection [31]. Zhang et al. designed a cascade amplification method based on CHA and HCR for miRNA detection [32]. Zhang et al. proposed a multi-layers DNA tetrahedron-assisted CHA for miRNA detection [33]. Although the sensitivity of these strategies were improved, their may suffer from some disadvantages: (i) multiple steps results in prolonged and tedious procedure; (ii) time-consuming process of multiple metastable hairpin-driven amplification and rigorous reaction conditions of enzyme-assist metastable hairpin-driven amplification restrict their practical applications; and (iii) the incompatible reaction conditions led to serious signal leakage.

**Scheme 1:**
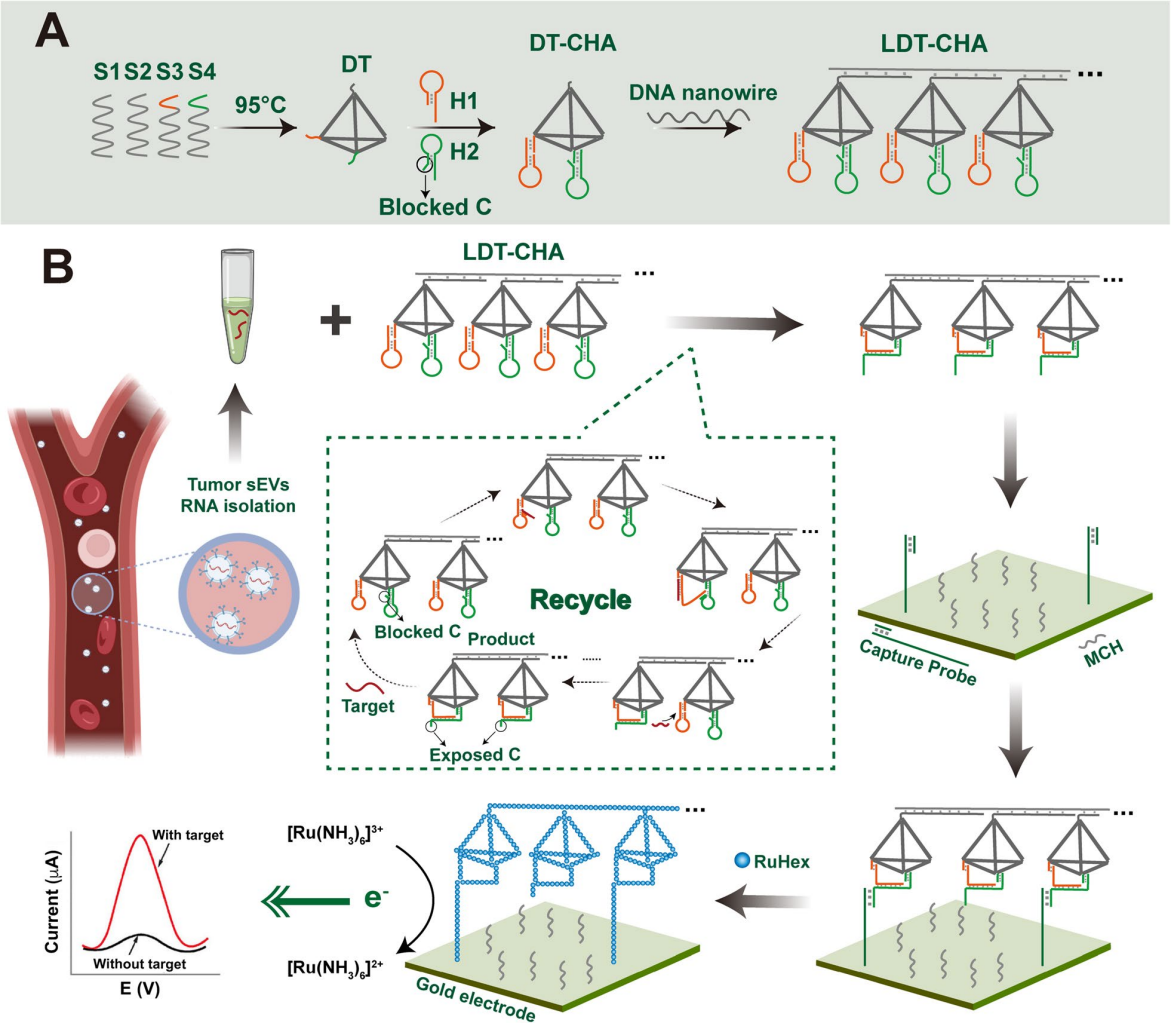
Schematic principle of the electrochemical biosensor for sEV-miRNAs determination. **A** Assembly process of LDT-CHA. **B** The reaction of LDT-CHA on the surface of electrode

Herein, we designed a novel electrochemical platform based on localized DNA tetrahedron-assisted catalytic hairpin assembly (LDT-CHA) for sensitive sEV-miRNA detection. Compared with other metastable hairpin-driven amplification strategies, our methods have multiple advantages. Firstly, DNA tetrahedron-assisted catalytic hairpin assembly (DT-CHA) could increase the local concentrations of CHA substrates by confining hairpins in a compact space, which dramatically improve the amplification efficiency. Secondly, the LDT-CHA contained a number of DT-CHAs as successive reactants in a confined space, which could minimize leakage of CHA by the precise-control space of each DT-CHA. Thirdly, The LDT could act as programmed track, once the driving motor is triggered by a target sEV-miRNA, the sEV-miRNA would move along the LDT-CHA to produce cascaded signal output. As a proof-of-concept, sEV-miR-1246 was employed as a model molecule because of its high expression in gastric tumour sEVs[34, 35]. The LDT-CHA was associated with DNA nanowires, DNA tetrahedrons, and the CHA system (H1 and H2). The DNA tetrahedrons contained four DNA strands (S1–S4). The 3′ sticky ends of S3 and S4 were adopted to anchor with CHA reactants (Scheme 1A). First, the sticky end C in H2, which could bind to the capture probe, was blocked. The LDT-CHA was established by hybridization of DNA tetrahedron-CHA (DT-CHA) to a DNA nanowire with repeated sequence. The sEV-miRNAs hybridized with one H1 in DT-CHA to trigger the CHA along the DNA nanowire due to the arrangement of DNA tetrahedrons in LDT-CHA and the programmed space. Next, multiple sticky end Cs were exposed by H1-H2 assembly on the tetrahedron and hybridized with the capture probe. Then, an amplified signal for the determination of sEV-miRNAs was generated by the electroactive substance RuHex, which links with the captured tandem tetrahedrons by electrostatic interactions (Scheme 1B). The proposed platform based on LDT-CHA holds great potential for sEV-miRNA detection and clinical application for cancer diagnostics.

## Characterization of the LDT-CHA

**Fig. 1:**
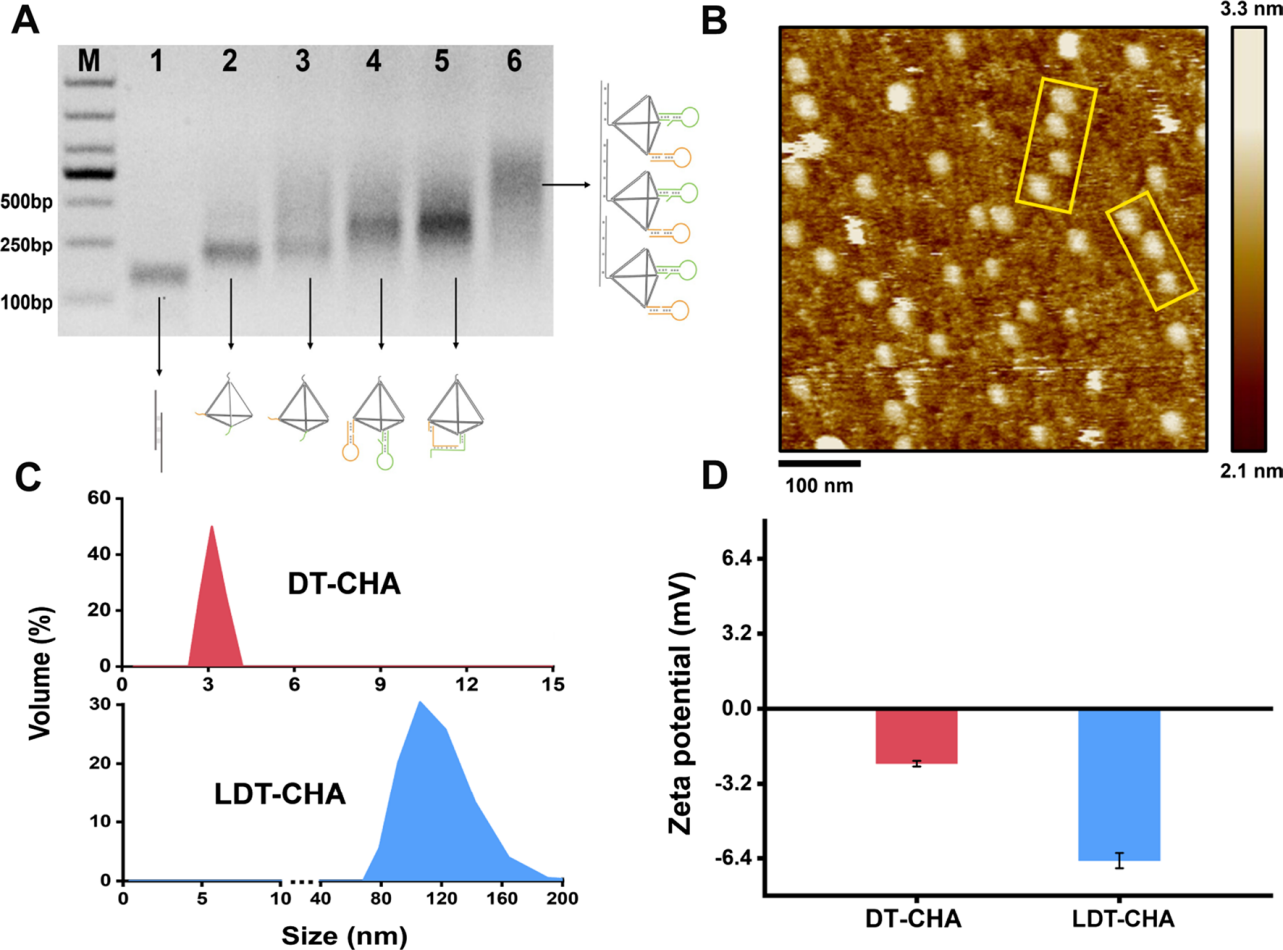
Characterization of the LDT-CHA. **A** Gel electrophoresis analysis of LDT-CHA assembly process. Lane M: 1000 bp marker; Lane 1: S1/S2; Lane 2: S1/S2/S3; Lane 3: S1/S2/S3/S4; Lane 4: S1/S2/S3/S4/H1/H2; Lane 5: S/S2/S3/S4/H1/H2/miR-1246; Lane 6: S1/S2/S3/S4/H1/H2/nanowire. **B** Atomic force microscope imaging of LDT-CHA. **C** Dynamic light scattering of DT-CHA and LDT-CHA particle size. **D** Zeta potential analysis of DT-CHA and LDT-CHA nanoparticles

The successful assembly of LDT-CHA was first confirmed by agarose gel electrophoresis. As shown in Fig. 1A, the probes were combined into DNA tetrahedron (Lane 1 to 3), DT-CHA (Lane 4), and LDT-CHA (Lane 6). Notably, in the presence of target miRNA, a more complex spatial structure of DT-CHA was generated, and the electrophoretic mobility was reduced (Lane 5). Atomic force microscopy (AFM) was used to reveal the morphologies of the LDT-CHA, showing a larger ordered arrangement of linked patterns (Fig. 1B, Additional file 1: Fig. S1) than that of the DT-CHA (Additional file 1: Fig. S2). The molecular size increased from 3 nm for DT-CHA to 105 nm for LDT-CHA, as suggested by DLS (Fig. 1C). Compared with DT-CHA, the zeta potential distribution of LDT-CHA decreased from − 2.3 to − 6.5 mV because of the enhanced negative electricity of the localized DT-CHA assembly (Fig. 1D). These results demonstrated that LDT-CHA was successfully prepared with high yield.

## The feasibility of LDT-CHA

**Fig. 2:**
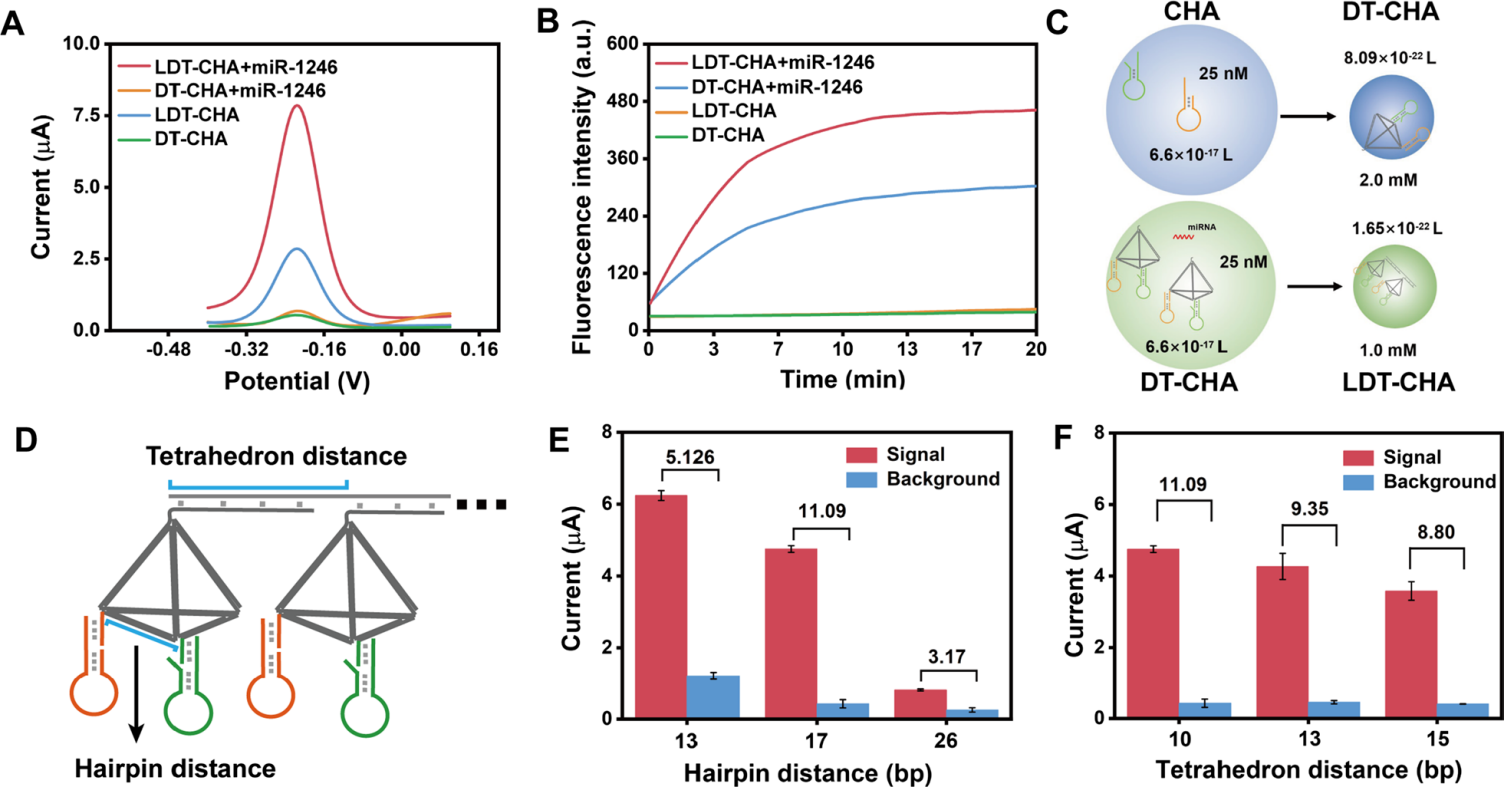
**A** DPV analysis and **B** Fluorescence kinetics monitoring of LDT-CHA and DT-CHA. **C** Comparison of the reaction space and local concentration of traditional CHA and DT-CHA (upper), DT-CHA and LDT-CHA (lower). **D** The scheme of LDT-CHA with different distance of hairpins and Tetrahedrons. **E** Signal-to-noise ratio of the LDT-CHA with different hairpins distance in the presence of 10 nM miR-1246. **F** Signal-to-noise ratio of the LDT-CHA with different tetrahedrons distance in the presence of 10 nM miR-1246

The superior features of LDT-CHA were first confirmed by DPV measurement. Compared with DT-CHA, the DPV signal of LDT-CHA was improved almost threefold, and the background signal was almost unchanged (Fig. 2A). This means that LDT-CHA obtained higher sensitivity and a wider linear range than DT-CHA for sEV-miRNA analysis. We used fluorescence signal measurements to further confirm the amplification of LDT-CHA. The hairpin DNA H1 modified with energy donor FAM and receptor BHQ1 at both ends is designed to release fluorescence in response to the target. As shown in Fig. 2B, the time-dependent fluorescence signal substantially increased with the addition of miRNA-1246. The fluorescence plateau of LDT-CHA was obtained in 10 min, and the signal of LDT-CHA was almost 2 times higher than that of DT-CHA, which is consistent with the electrochemical outcomes. These results demonstrated the high sensitivity and minimized leakage of LDT-CHA.

The outstanding sensitivity of LDT-CHA was confirmed based on collision theory (V = 1/cN), in which N represents the Avogadro constant, C represents the concentration of reactants, and V represents the local sphere volume. When the solution of the traditional CHA system included 25 nM H1 and 25 nM H2, the volume of collision space was calculated to be 6.6 × 10^–17^ L (Fig. 2C). After CHA was tied to the DNA tetrahedron (DT-CHA), the distance between H1 and H2 was shortened to 5.78 nm (17 bp). Then, the local concentrations of H1 and H2 were both elevated to 2.0 mM. Similarly, supposing that the solution contains 25 nM DT-CHA, the distance between DT-CHA was reduced to 3.74 nm. According to the analytical formula V = 1/cN, the space for the two DT reaction units was 1.65 × 10^–17^ L, and the local concentration was elevated to 1.0 mM. The direct correlation of collision frequency to hairpin concentration indicates that the enhanced reaction kinetics of LDT-CHA were caused by the increased local concentration.

The balance between the localized reaction and leakage reaction plays a vital role in LDC because of the DNA breath reaction. In the LDT-CHA system, the CHA breath reaction depends on the distance between hairpins (H1 and H2), which are connected by a DNA tetrahedron (Fig. 2D). To study the effect of the distance between H1 and H2 on the DNA tetrahedron, three DNA tetrahedrons with distances of 13, 17 and 26 base pairs (13-DT, 17-DT, and 26-DT) were synthesized. As shown in Fig. 2E, the 17 bp TDs had the best performance in the signal-to-noise ratio. In contrast, the 13 bp TD had a high background signal, and the 26 bp TD had low efficiency for the CHA reaction. Thus, the 17 bp TD was chosen as the best design. To study the effect of distance between DNA tetrahedrons, three different distances were designed (Fig. 2F). The 10 bp distance had a high efficiency in the signal-to-noise ratio because the steric hindrance of the CHA system was reduced by the three-dimensional structure of the DNA tetrahedron.

## Characterization of the electrochemical biosensor

**Fig. 3:**
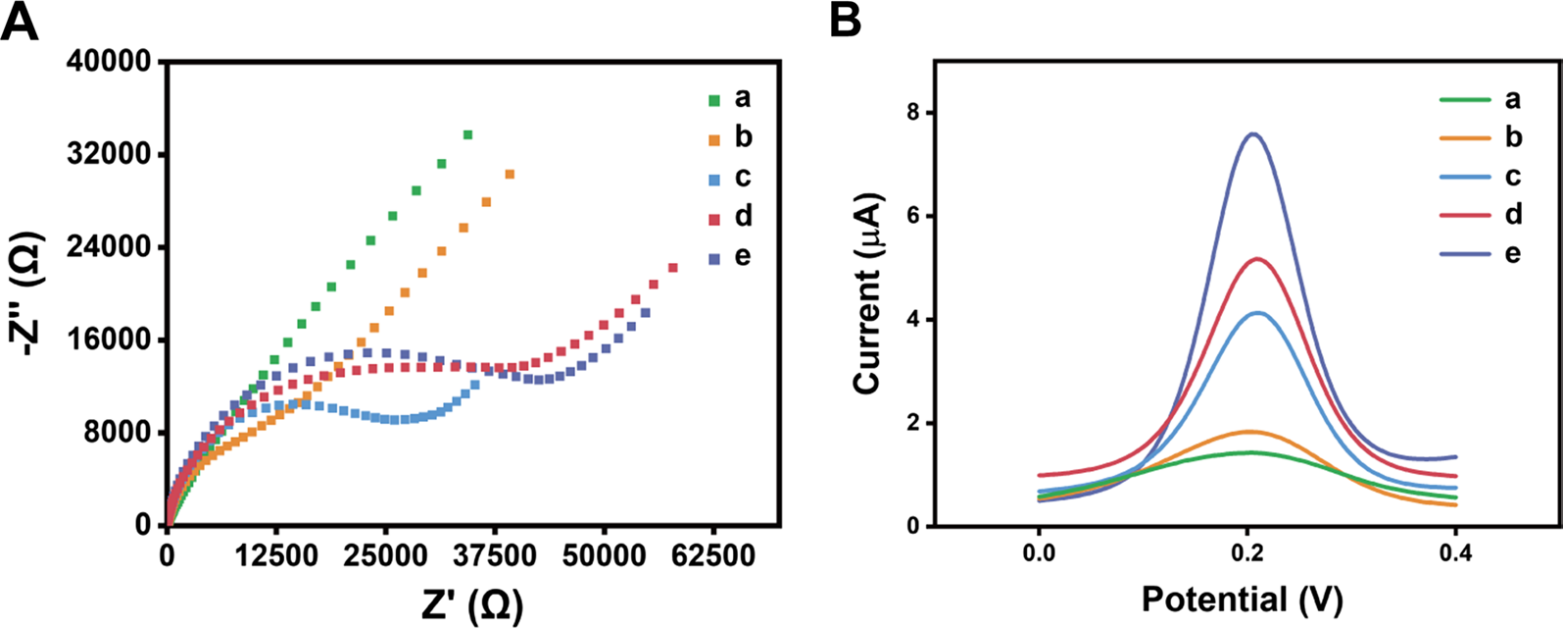
**A** EIS and **B** SWV of the bare gold electrode (**a**); the electrode modified with capture probe (**b**); the modified electrode after MCH block (**c**); DT-CHA captured by the prepared electrode (**d**); LDT-CHA captured by the prepared electrode (**e**)

To confirm the assembly of LDT-CHA on the electrode surface, we characterized the electrochemical biosensor by EIS and SWV analysis. In the EIS measurements, the diameter of the semicircle on the curve reflects the electron transfer resistance proportionally (Ret). As demonstrated in Fig. 3A, the bare gold surface line was almost straight (curve a), indicating the good electrical conductivity of the gold electrode with low Ret. We next modified the gold electrode with double-strand capture probes via Au–S intermolecular interactions, resulting in larger DNA backbone aggregation. The [Fe(CN)_6_]^3−/4−^molecules were alienated to the electrode surface, resulting in an increase in Ret (curve b). Then, MCH was used to block the redundant sites of the electrode, which greatly increased Ret due to the steric effect of the large molecule (curve c). With the addition of target miRNA, the LDT-CHA combined the capture probe on the surface of the electrode showed greater Ret (curve e) than that of DT-CHA (curve d). SWV (Fig. 3B) analyses also demonstrated the step-by-step assembly of the electrode, which is consistent with the EIS results. These results indicate the successful assembly of the proposed biosensor and the powerful amplification of LDT-CHA.

## Analytical performance of the biosensor

**Fig. 4:**
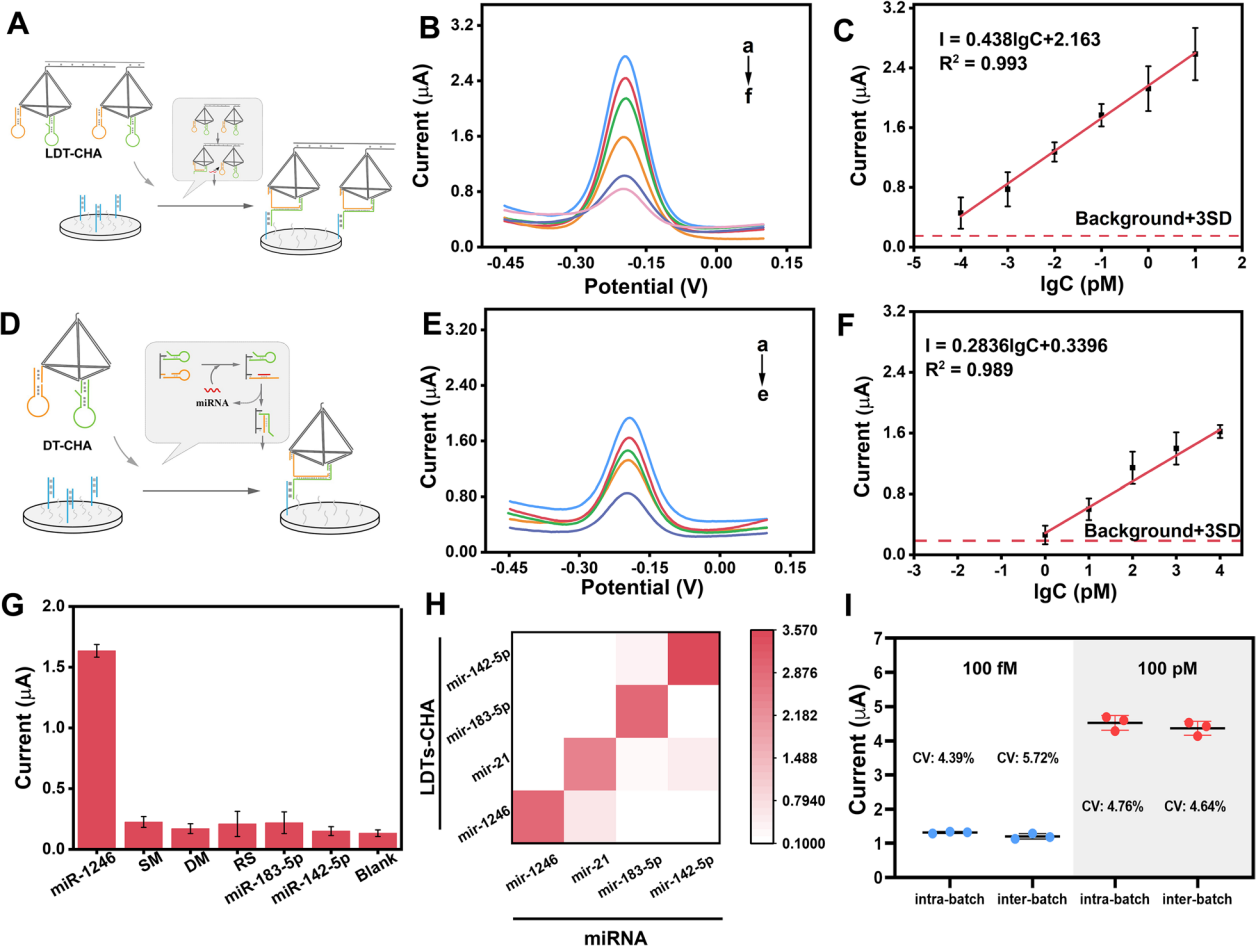
**A** Schematic illustration of the LDT-CHA strategy. **B** DPV responses and **C** corresponding calibration curve of target miRNA with the concentration from 100 aM to 10 pM (a: 10 pM; b: 1 pM; c: 0.1 pM; d: 0.01 pM; e: 0.001 pM; f: 0.0001 pM) using LDT-CHA. **D** Schematic illustration of the DT-CHA strategy. **E** DPV responses (**F**) and corresponding calibration curve (**F**) of target miRNA with the concentration from 1 fM to 10 nM (a: 10 nM; b: 1 nM; c: 100 pM; d: 10 pM; e: 1 pM) using DT-CHA. **G** DPV results respond to target miRNA and five interferences. **H** Heat map analysis of the orthogonal identification of four sEV-miRNAs. **I** Stability of the LDT-CHA. The error bars are the standard deviations by three repetitive assays (n = 3)

Before demonstrating the properties of the proposed biosensor, some key conditions were optimized, as shown in Additional file 1: Figs. S3 and S4. Then, we studied the analytical performance, including sensitivity, specificity, and reproducibility, using DPV measurements. To exhibit the superior sensitivity of LDT-CHA, the DPV signals of LDT-CHA (Fig. 4A), DT-CHA (Fig. 4D), and CHA (Additional file 1: Fig. S5) in response to sEV-miR-1246 were detected. With increasing sEV-miR-1246 concentrations, the DPV signals of LDT-CHA (Fig. 4B) were greatly enhanced compared to those of DT-CHA (Fig. 4E) and CHA (Additional file 1: Fig. S5B). Moreover, the LDT-CHA had a broad linear range from 100 aM to 10 pM with a correlation equation fitted as I = 0.438 lgC_target_ + 2.163 (R^2^ = 0.993) (Fig. 4C). Based on the 3SD principle, the limit of detection of LDT-CHA was obtained at 21 aM, which was 7790-fold lower than that of DT-CHA (Fig. 4F) and 173616-fold lower than that of CHA (Additional file 1: Fig. S5C), demonstrating the enhanced sensitivity of the LDT-CHA strategy.

The specificity of the platform was measured using different groups of sEV-miRNAs containing a single-base mismatch sequence (SM), double-base mismatch sequence (DM), random sequence (RS), and several homologous sEV-miRNAs (sEV-miR-21, sEV-miR-183-5P, and sEV-miR-142-5P). As exhibited in Fig. 4G, the electrochemical signal of miR-1246 was almost 10 times higher than those of mismatched sequences and other sEV-miRNAs, indicating the high specificity of the electrochemical platform. The specificity of LDT-CHA was confirmed further using orthogonal identification of four sEV-miRNAs of LDT-CHA. As shown in Fig. 4H, only the appropriate LDT-CHA response to correct sEV-miRNAs generated an enhanced signal. To explore the reproducibility of the proposed biosensor, the samples containing 100 fM and 100 pM target miRNA were tested in regards to interbatch and intrabatch variability, respectively. The variable coefficient of the result was approximately 6% (Fig. 4I), indicating a suitable reproducibility of this platform. Next, to evaluate the stability of the proposed platform, the biosensor was measured in the presence of 10 nM miRNA-1246 for 8 days. The electrochemical signals of the initial measurement were maintained at 85.4% (Additional file 1: Fig. S6).

## Determination of sEV-miRNAs in cell lines

**Fig. 5:**
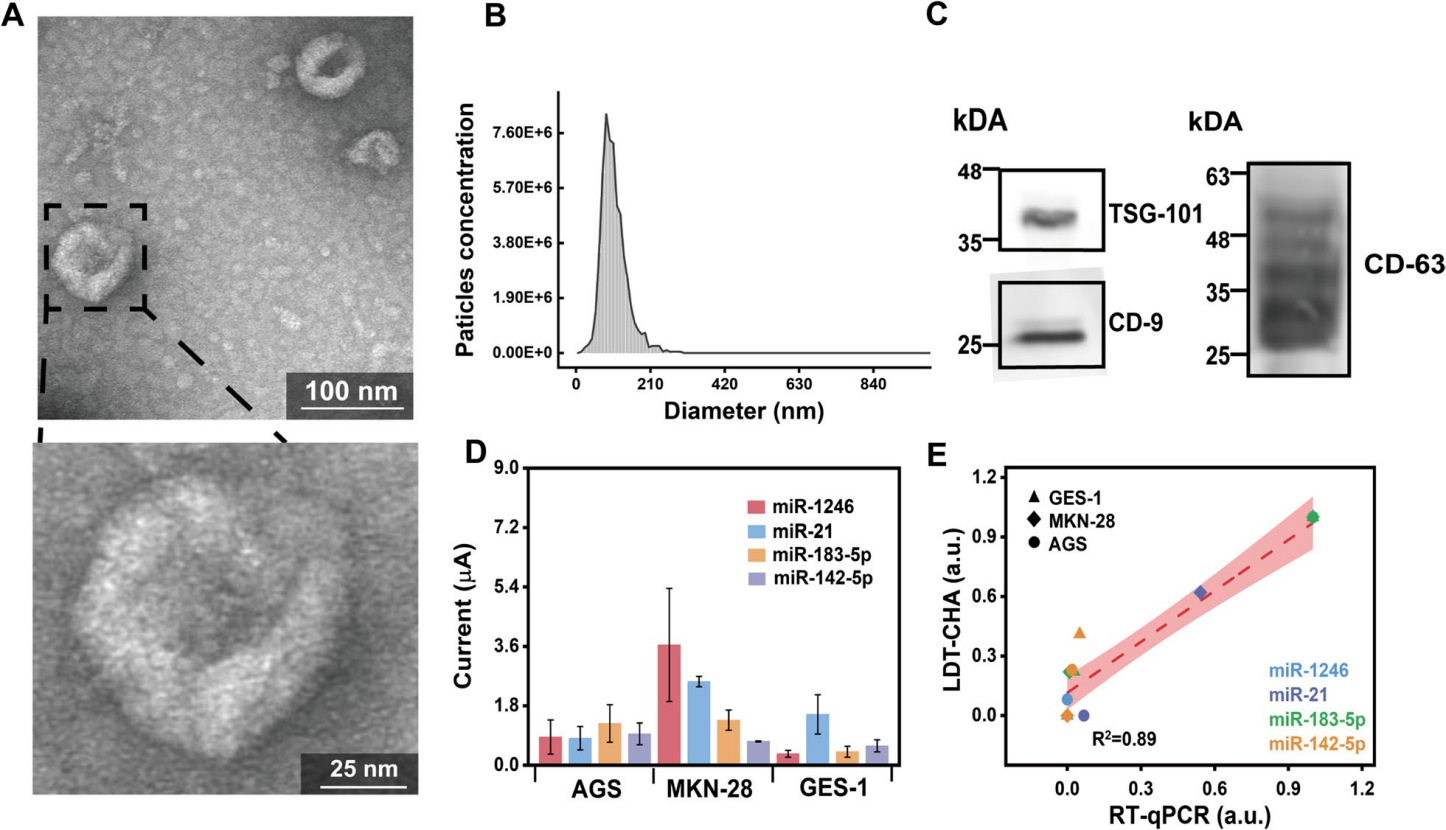
**A** TEM images of model sEV isolated from MKN-28 cell lines (scale bar: 100 nm (upper) and 50 nm (lower)). **B** Size distribution analysis of MKN-28 cell sEV. **C** The expression of specific sEV proteins by Western blot analysis. **D** DPV measurements for four sEV-miRNAs expression in sEV derived from MKN-28, AGS, and GES-1 cell lines. **E** The correlation between LDT-CHA and RT-qPCR method for detecting four sEV-miRNAs expression in sEV derived from MKN-28, AGS, and GES-1 cell lines. The bright red-dashed line indicates the best linear fit in the log–log scale, and the shaded red area represents the 95% confidence

Before the determination of the sEV-miRNAs, the standard ultracentrifugation method was adopted for separating EVs derived from a gastric tumour cell line (MKN-28). The represented EV derived from the MKN-28 cell line was characterized by a standard protocol. Typical partially collapsed discs with diameters of 110 nm are shown in TEM images (Fig. 5A). The NTA exhibited a group ranging from 51 to 208 nm with a peak at 110 nm (Fig. 5B). The specific protein markers of EVs (positive proteins: CD9, CD63, and TSG101) were confirmed by Western blotting analysis (Fig. 5C). These results indicated the high purity and integrity of the isolated EV.

Then, the sEVs derived from two gastric tumour cell lines (MKN-28 and AGS) and a normal gastric epithelial cell line (GES-1) were separated to extract total RNA. The proposed platform was adopted to measure the panel of four gastric tumour-associated sEV-miRNAs (sEV-miR-1246, sEV-miR-21, sEV-miR-183-5p, and sEV-miR-142-5p). As shown in Fig. 5D, the expression levels of the four sEV-miRNAs in various EVs were different, and most of them were much higher in sEVs from gastric tumour cell lines than in those from the normal epithelial cell line, especially those of sEV-miR-1246 and sEV-miR-183-5p. qRT–PCR was adopted to further determine the expression levels of the sEV-miRNAs and compare the obtained results with those obtained by the electrochemical platform, which were consistent with those of our developed biosensor (R^2^ = 0.89) (Fig. 5E). These results indicate the application potential and accuracy of the developed electrochemical biosensor.

## Clinical sample analysis

**Fig. 6:**
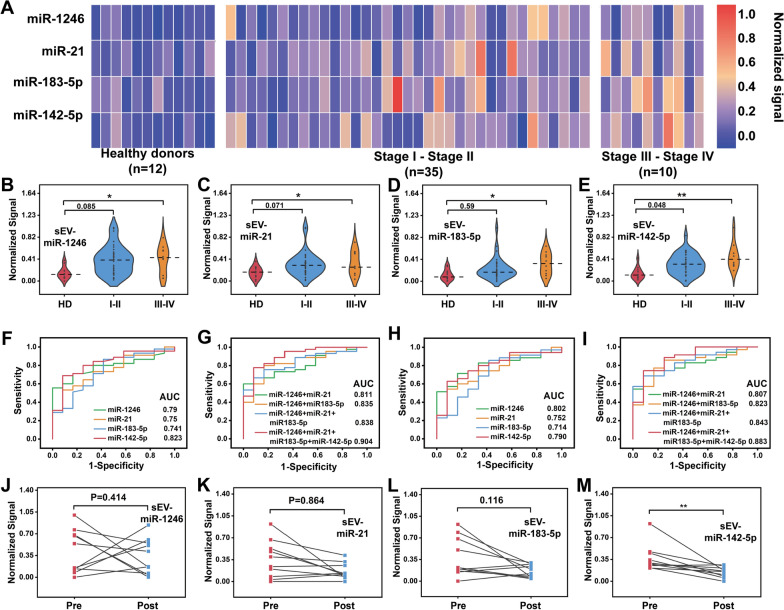
**A** The expression of four sEV-miRNAs measured by the electrochemical platform in gastric cancer cohort (healthy donors, n = 12; patients with gastric cancer in stage I–II, n = 35; stage III–IV, n = 10. **B**–**E** The electrochemical signal for sEV-miR-1246, sEV-miR-21, sEV-miR-183-5p and sEV-miR-142-5p profiling in gastric cancer cohort (*p < 0.1; **p < 0.05). **F**, **G** ROC curve of single sEV-miRNA and combined sEV-miRNAs for diagnosing patients with gastric tumors. **H**, **I** ROC curve of single sEV-miRNA and combined sEV-miRNAs for identifying gastric cancer in early stages (I–II). **J**–**M** Plasma samples from gastric cancer patients (n = 9) before (Pre) and after (Post) surgery measured by sEV-miRNAs profiles (**J** sEV-miR-1246; **K** sEV-miR-21; **L** sEV-miR-183-5p; and **M** sEV-miR-142-5p; **p < 0.05)

To explore the clinical applications of the proposed biosensor, a gastric cancer patient cohort (n = 57; 35 patients with stage I-II disease, 10 patients with stage III-IV disease, and 12 healthy donors as controls) was developed (Additional file 1: Table S2). sEV-miRNA was isolating after EVs separation by ultracentrifugation. The sEV-miRNA expression profiles are summarized in Fig. 6A. As shown in Fig. 6B–E, the average levels of all four sEV-miRNAs were highest in patients with stage III-IV disease, and sEV-miR-142-5p had the best capacity for distinguishing gastric cancer from healthy donor samples (p < 0.05). However, the two distributions of the patients with stage I-II disease and healthy donors overlapped, which made identification based on a single sEV-miRNA difficult. To improve the clinical performance of the biosensor for cancer diagnosis, we proposed using a combination of sEV-miRNAs and analysing the ROC curves. Compared to the single sEV-miRNA (Fig. 6F and H), the combination of four sEV-miRNAs showed higher accuracy (AUC: 0.904, 95% CI 0.808 to 0.999) for discriminating patients with gastric cancer from healthy donors (Fig. 6G and Additional file 1: Fig. S7). Moreover, the combination of four sEV-miRNAs showed a sensitivity of 74.3% and a specificity of 66.0% (AUC: 0.883, 95% CI 0.772 to 0.995) for the identification of gastric cancer with stages I-II (Fig. 6I and Additional file 1: Fig. S8).

To further demonstrate the clinical applicability of this biosensor, the sEV-miRNA profile changes were tracked in patients who underwent clinical treatment (Additional file 1: Table S3). We compared sEV-miRNA expression before and after the operation. The expression levels of sEV-miR-1246 (Fig. 6a), sEV-miR-21 (Fig. 6b), and sEV-miR-183-5p (Fig. 6d) showed no significant changes, with P values of 0.414, 0.864, and 0.116, respectively (paired t test). In contrast, sEV-miR-142-5p decreased in all patients (Fig. 6c) (P < 0.01, paired t test); thus, it may be used as a candidate marker for treatment monitoring.

## Discussion

The sEV-miRNA in the circulation is promising biomarkers for cancer diagnostics. However, most methods for sEV-miRNA measurement require sophisticated operation and expensive equipment. Here, we present a rapid and sensitive electrochemical biosensor based localized DNA carrier and catalytic hairpin assembly for rapid determination of multiple sEV-miRNAs with high sensitivity and selectivity in clinical samples. For the nucleic acid amplification of LDT-CHA, The DNA nanowire confined DT-CHA in a compact space accelerating the CHA amplification by DNA tetrahedron and minimizing leakage of the localized reaction by creating a precise-control space for each DT-CHA, demonstrating superior sensitivity. In this work, LDT-CHA, an metastable hairpin-driven amplifier based on localized DNA tetrahedrons and catalytic hairpin assembly, has been adopted to detect the low expression sEV-RNAs in serum of GC patients. Furthermore, the amplification can be completed in one step, which greatly simplified the operation. By our unique combination of the localized DNA tetrahedrons with the CHA, this platform can measure the target sEV-miRNA down to a concentration of 25 aM within 30 min with high specificity and repeatability. Compared to a previous platform based on metastable hairpin-driven amplification (Additional file 1: Table S4), the proposed electrochemical biosensor based on LDT-CHA has better sensitivity with a short processing time. Although the surface-enhanced Raman-based method has higher sensitivity, its applications are restricted by the tediousness of the process and expensiveness of the equipment.

Moreover, the results we detected by LDT-CHA of sEV-miRNA show difficulty distinguishing GC patients. Thus, multiple sEV-miRNA are necessary for GC diagnosis due to the heterogeneous feature of sEV from patients. Using a four-sEV-miRNA panel, the accuracy of this biosensor was 90% for the discrimination of cancer versus healthy individuals in a clinical cohort (n = 57). The sensitivity, specificity and accuracy to distinguish gastric cancer patients at an early stage are 74, 66 and 88%, respectively. It is worth noting that the sEV-miRNA also demonstrate the potential for effective GC prognostic evaluation, which could further expand clinical applications of this platform. Our next step is to expand the panel of sEV-miRNA and conduct a large-scale validation study to improve the accurate of clinical applications. This platform holds the potential to combined with other strategies, such as separation and microfluidic technology, to realize sEV isolation, signal amplification, and simultaneous detection of multiple sEV-miRNAs on one system, producing a high efficiency ‘sample in-result out’ biosensor.

Although LDT-CHA need to prepare in advance, it does not need complex operation, sophisticated instrument, or trained personnel, which are required for most of the sEV-miRNA analysis methods at present. Moreover,the total cost of the platform can be as low as $0.32 per test (Additional file 1: Table S5). Overall, the proposed biosensor with features including simple operation, rapid testing, and inexpensive equipment, which will advance point-of-care testing in the field of noninvasive diagnosis and cancer monitoring.

## Supplementary Information

**Additional file 1.** Materials and methods. Figure S1. 3D image of LDT-CHA characterization by atomic force microscopy (AFM). Figure S2. (A) 2D and (B) 3Dimage of DT-CHA characterization by atomic force microscopy (AFM); Scale bars are 1 um. Figure S3. The effect of the reaction time of LDT-CHA. Figure S4. The effectof the incubating temperature of LDT-CHA. Figure S5. (A) Schematic illustration of traditional CHA. (B) DPV respond and corresponding calibration curve from 1 pM to1 nM using traditional CHA. (C) Corresponding calibration curve of target miRNA with the concentration from 10 pM to 100 nM using Traditional CHA, error bars representstandard deviations of the measurements (n = 3). Figure S6. The stability of the proposed LDT-CHA platform, error bars represent standard deviations of the measurements (n = 3). Figure S7. ROC curve of combined sEV-miRNAs for identifying patients with gastric tumors. Figure S8. ROC curve of combined sEVmiRNAsfor identifying patients with early-stage gastric tumors. Table S1. DNA sequences of used in this assay. Table S2. Clinical information for healthy donors (HD) and patients with gastric cancer (GC). Table S3.Clinical information for patients who underwent clinical treatment comparison. Table S4. Comparison of different biosensors for detecting sEV-miRNA. Table S5. Cost analysis.

## Data Availability

Not applicable.
