# Supplementary material for: Localized DNA tetrahedrons assisted catalytic hairpin assembly for the rapid and sensitive profiling of small extracellular vesicle-associated microRNAs
Source: J Nanobiotechnology. 2022 Dec 1;20:503. doi: 10.1186/s12951-022-01700-6 (PMC9714172; doi:10.1186/s12951-022-01700-6)
Supplement: Supplementary file 1 — Additional file 1. Materials and methods. Figure S1. 3D image of LDT-CHA characterization by atomic force microscopy (AFM). Figure S2. (A) 2D and (B) 3Dimage of DT-CHA characterization by atomic force microscopy (AFM); Scale bars are 1 um. Figure S3. The effect of the reaction time of LDT-CHA. Figure S4. The effectof the incubating temperature of LDT-CHA. Figure S5. (A) Schematic illustration of traditional CHA. (B) DPV respond and corresponding calibration curve from 1 pM to1 nM using traditional CHA. (C) Corresponding calibration curve of target miRNA with the concentration from 10 pM to 100 nM using Traditional CHA, error bars representstandard deviations of the measurements (n = 3). Figure S6. The stability of the proposed LDT-CHA platform, error bars represent standard deviations of the measurements (n = 3). Figure S7. ROC curve of combined sEV-miRNAs for identifying patients with gastric tumors. Figure S8. ROC curve of combined sEVmiRNAsfor identifying patients with early-stage gastric tumors. Table S1. DNA sequences of used in this assay. Table S2. Clinical information for healthy donors (HD) and patients with gastric cancer (GC). Table S3.Clinical information for patients who underwent clinical treatment comparison. Table S4. Comparison of different biosensors for detecting sEV-miRNA. Table S5. Cost analysis. [file 12951_2022_1700_MOESM1_ESM.docx]

**Localized DNA tetrahedrons assisted catalytic hairpin assembly for the rapid and sensitive profiling of small extracellular vesicle-associated microRNAs**

*Ye Zhang^12^*^‡^**, Wenbin Li^12^*^‡^*, Tingting Ji^12^*^‡^*, Shihua Luo^5^*^‡^*, Jiuxiang Qiu^14^, Bo Situ^12^, Bo Li^12^, Xiaohe Zhang^12^, , Tiange Zhang^12^, Wen Wang^12^, Yunju Xiao^6^, Lei Zheng^123^*, Xiaohui Yan^12^**

^1^Laboratory Medicine Center, Department of Laboratory Medicine, Nanfang Hospital, Southern Medical University, Guangzhou 510515, China

^2^Guangdong Engineering and Technology Research Center for Rapid Diagnostic Biosensors, Nanfang Hospital, Southern Medical University, Guangzhou 510515, China

^3^Department of Clinical Laboratory, Shunde Hospital, Southern Medical University (the First People’s Hospital of Shunde), Foshan 528300, Guangdong Province, China

^4^Department of Laboratory Medicine, Guangzhou Eighth People’s Hospital, Guangzhou Medical University, Guangzhou 510515, China

^5^Center for Clinical Laboratory Diagnosis and Research, the Affiliated Hospital of Youjiang Medical University for Nationalities, Baise 533000, Guangxi, PR China

^6^Division of Laboratory Medicine, Guangdong Provincial People's Hospital, Guangdong Academy of Medical Sciences, Guangzhou 510000, China

**Keywords:** DNA tetrahedron, catalyzed hairpin assembly, sEV-miRNAs, early cancer diagnostics

*Corresponding author at: Department of Laboratory Medicine, Nanfang Hospital, Southern Medical University, Guangzhou 510515, Guangdong Province, PR China. E-mail address: gzyanxh@126.com [(X.H Yan)](mailto:qyr@smu.edu.cn%20(Y.R%20Qiu)); nfyyzhenglei@smu.edu.cn (L. Zheng).

^‡^ These authors contributed equally to this work.

**Materials and methods**

**Reagents and instruments**

GL DNA marker and DNA loading buffer were purchased from Accurate Biology (Hunan, China). TE buffer and 4S Green Plus were obtained from Sangon Inc (Shanghai, China). All the DNA stands were synthesized and purified by Sangon (Shanghai, China) (Table. 1). Trizol reagent was supported by Fdbio science (Hangzhou, China). sEV isolation reagent was procured by Ribbio (Guangzhou, China). TNaK buffer contained 125 mM NaCl, 20 mM Tris-HCl, and 20 mM KCl. The Bst DNA polymerase used for PER was got from Tosanbio (Guangzhou, China). Binding buffer for PER system was prepared with 500 mL PBS and 0.55 mM MgCl_2_.

All the electrochemical tests included differential pulse voltammetry (DPV), impedance spectroscope (EIS), and square wave voltammetry (SWV) were analyzed by CHI660E workstation (Shanghai Chenhua Instruments Inc). The gel electrophoresis analysis was implemented at Syngene electrophoresis analyzer (Syngene, England). Ultracentrifuge was provided from Beckman Optima XPN-100 (Beckman, USA).

**Preparation of LDTs-CHA**

All DNA strands were heated on 95 °C for 5 min, then cooling slowly to room temperature, then stored at 4 °C for the following use. DNA nanowire was generated by Primer Exchange Reaction (PER). The PER reaction was performed by PER hairpin (100 nM), DNA primer (1 mM), Bst DNA polymerase (2 μL), MgCl_2_ (100 mM), binding buffer and Thermo buffer (1×) at 37 °C for 2 h, then heated on 85 °C lasting 20 min for inactivation. The final concentration of 25 nM DT, 10 nM PER product, 25 nM of H1 and H2 were assembled to form LDT-CHA.

**Nucleic acid electrophoresis**

To confirm the synthesis of LDT-CHA, 3 % agarose gels were used for analysis (1×TAE buffer, 140 V voltage, 50 min). The gel was visualized by Syngene G: BOX F3 imaging system (Syngene, England).

**Fluorescence measurement**

The total reaction contained 40 μL LDT-CHA and 20 μL TNaK buffer. The fluorescence dynamic monitoring was recorded every 30 s within 30 min by luminescence spectrometer (excitation wavelength: 488 nm; emission wavelength: 515 nm) (PerkinElmer LS-55, USA).

**Preparation of the electrochemical platform**

Electrode was polished using 0.05 alumina powder. Next, cleaning in ultrasound and washing with distilled water for 3 times to ensure a clean gold surface. Next, the gold electrode was dried by compressed air after purified with piranha solution. Adding 7 μL prepared capture probe (50 nM) into the electrode surface and stored at 4°C overnight, then 1 mM MCH was used to block the non-specific sites of electrode.

10 μL mixture of LDT-CHA and sEV-miRNAs was added to the surface of electrode for 30 min. After washing with cleaning buffer, 5 μM RuHex dissolved in Tris-HCl buffer was dripped on the electrode surface. Then, the DPV test was performed. All the preparation were operated at 25 °C

**Cell culture and preparation**

Human gastric cancer cell lines (MKN-28, AGS) were employed as positive cell line. Negative cell line (GES-1) was used as control cell. All cell lines were cultured in DMEM and 10% FBS (Procell Co.,Ltd), incubating with 1% Penicillin-Streptomycin solution at 37°C with 5% CO_2._

**Isolation sEVs and RNA extraction**

Clinical plasma samples were collected from healthy donors and patients with gastric cancer (NanFang Hospital, Southern Medical University) and stored in -80 °C before use. The plasma sample (1 mL) were centrifuged at 3,000×g for 20 min, then, 10,000×g for 30 min for removal of impurities. The sEVs were purify by ultracentrifugation after isolated by Ribbio sEV isolation reagent. Typical Chloroform method was used for the total RNA extraction[^1^](#_ENREF_1). Then, the extrated RNA was stored at -80 °C for further use.

**RT-qPCR analysis**

The All-in-One miRNA RT-qPCR Detection System 2.0 (Gene Copoeia, China) was employed to detect sEV-miRNAs. The cDNA was reverse transcript at 42 °C for 60 min, inactivated in 70 °C for 10 min. Then using the protocol of 95 °C (10 min), 40 cycles of 95 °C (2 s), 60 °C (20 s), 70 °C (10 s) in the platform of Light Cycle 480 П (Roche, USA), with let-7 as external reference to detect miRNAs.

**Supporting Figures.**


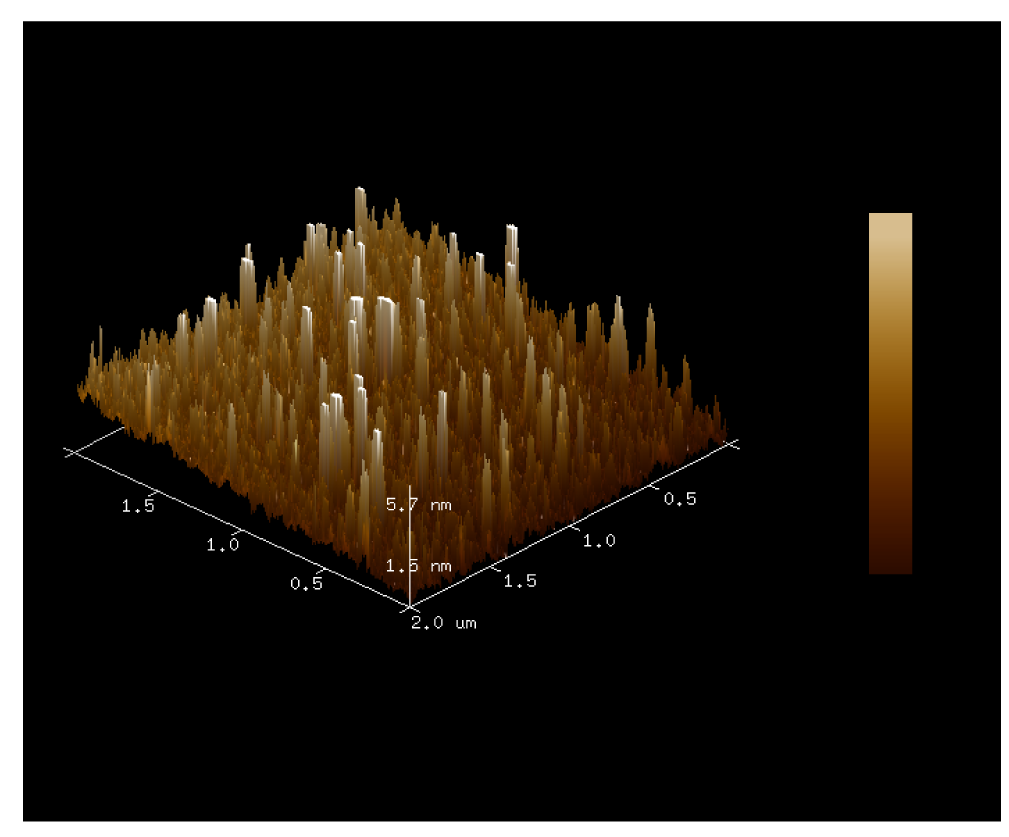


Figure S1. 3D image of LDT-CHA characterization by atomic force microscopy (AFM).


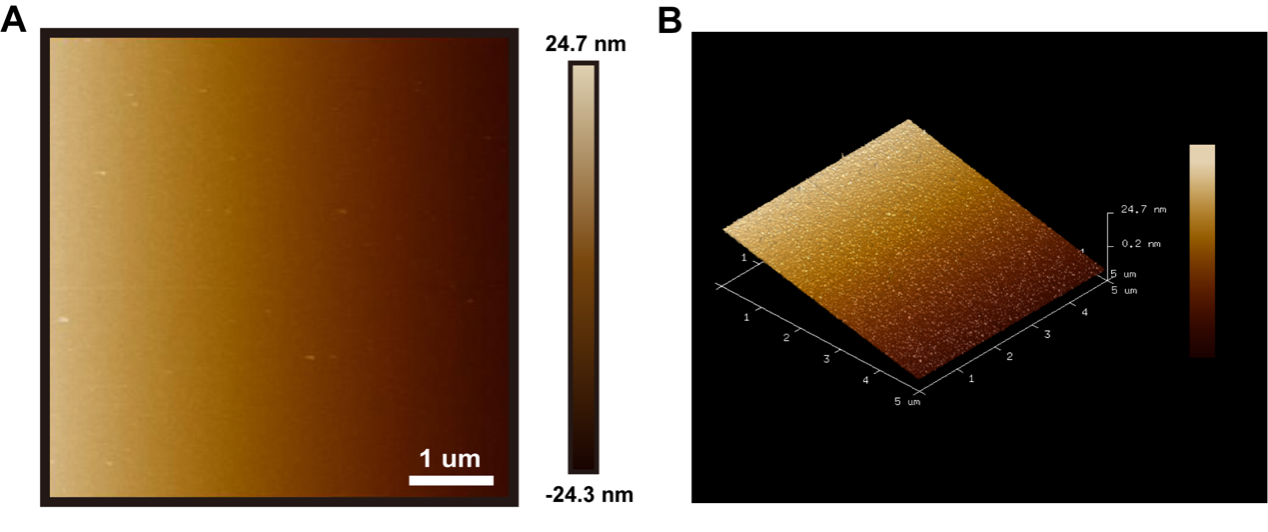
 Figure S2. (A) 2D and (B) 3D image of DT-CHA characterization by atomic force microscopy (AFM); Scale bars are 1 um.


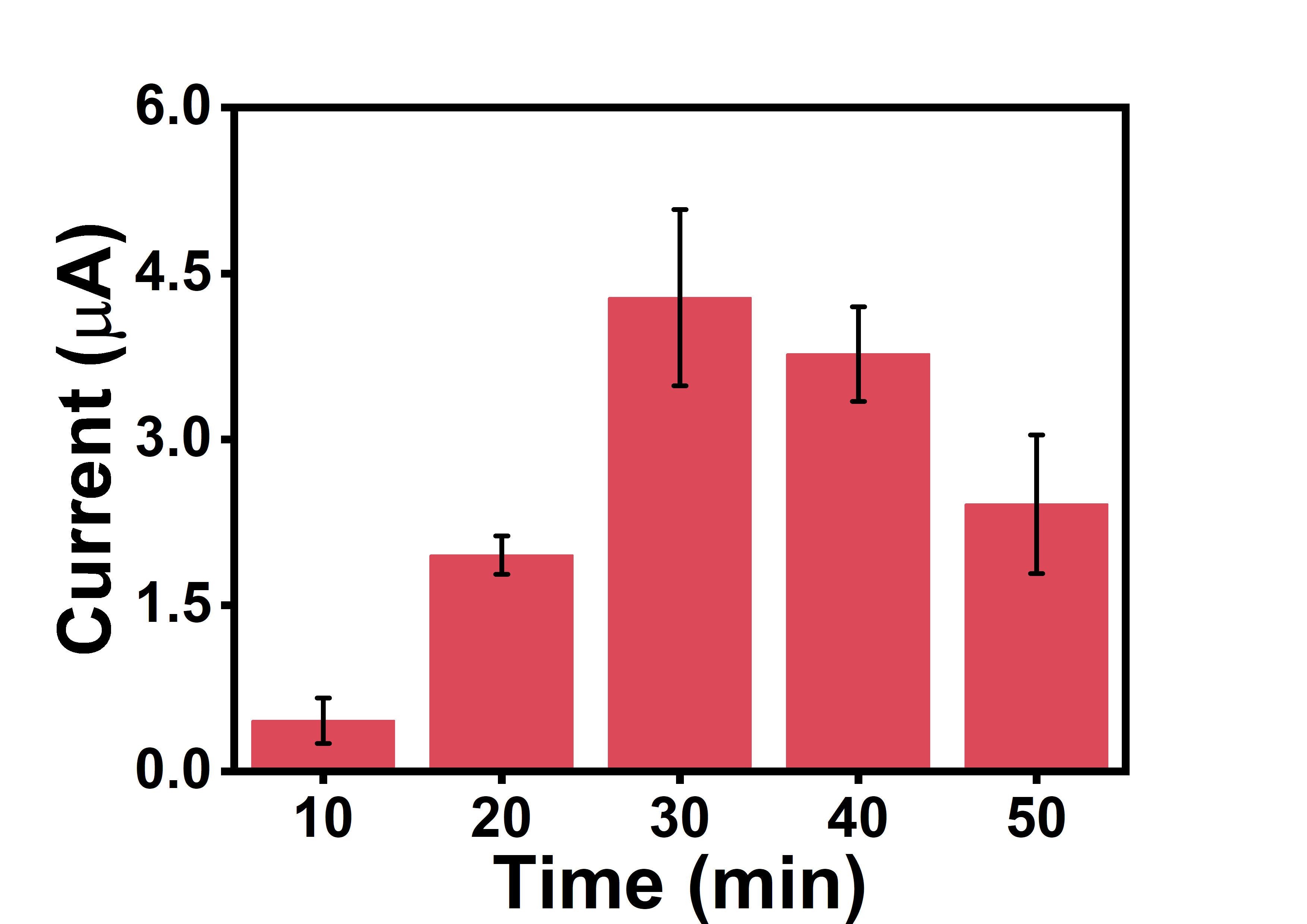


Figure S3. The effect of the reaction time of LDT-CHA.


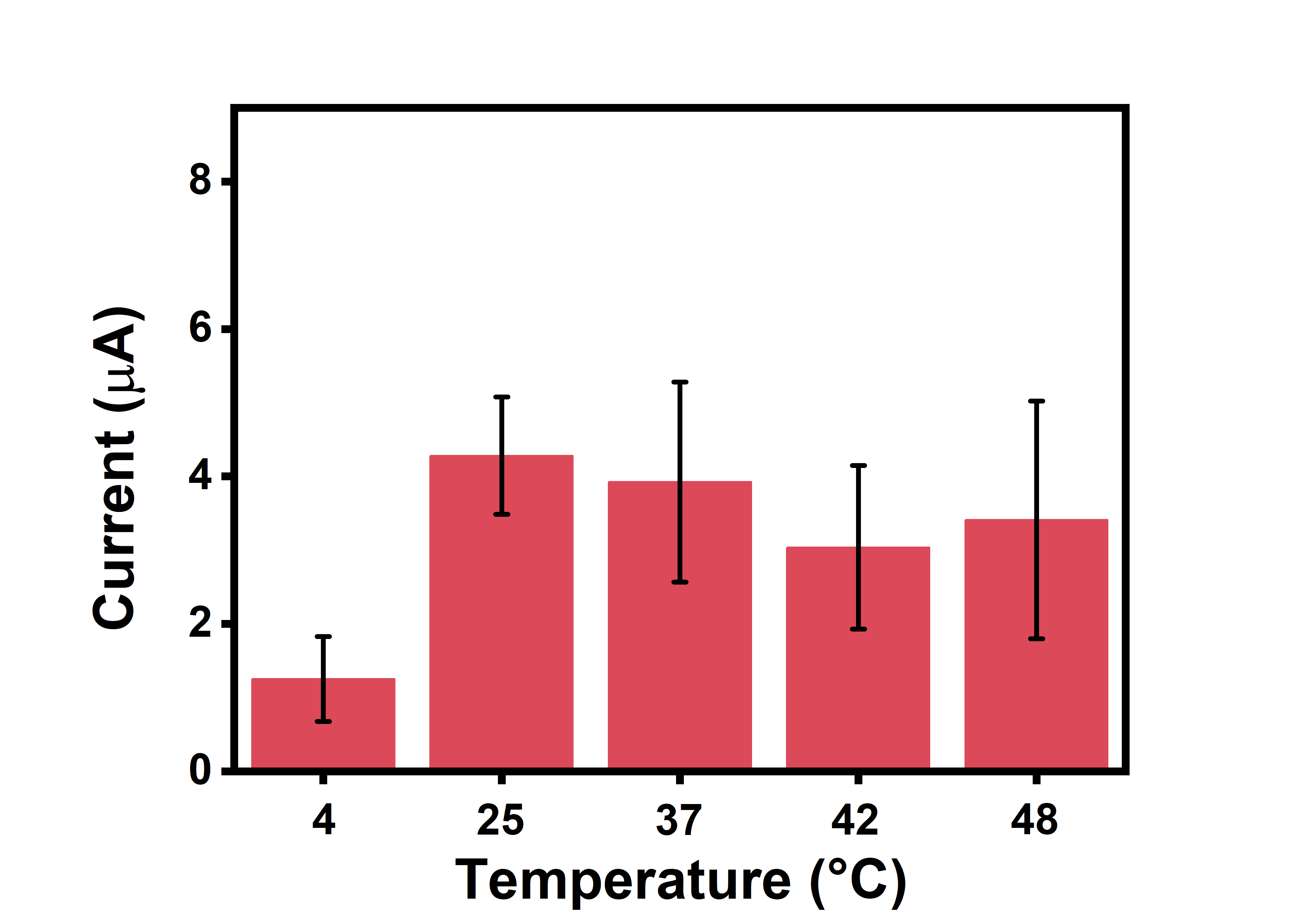


Figure S4 The effect of the incubating temperature of LDT-CHA.


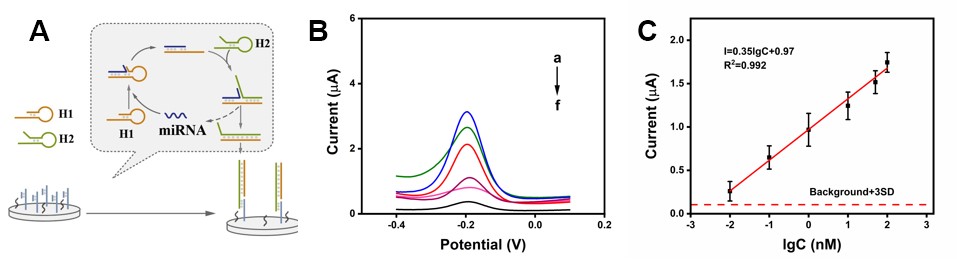


Figure S5. (A) Schematic illustration of traditional CHA. (B) DPV respond and corresponding calibration calibration curve from 1 pM to 1 nM using traditional CHA. (C) Corresponding calibration curve of target miRNA with the concentration from 10 pM to 100 nM using Traditional CHA,error bars represent standard deviations of the measurements (n = 3).


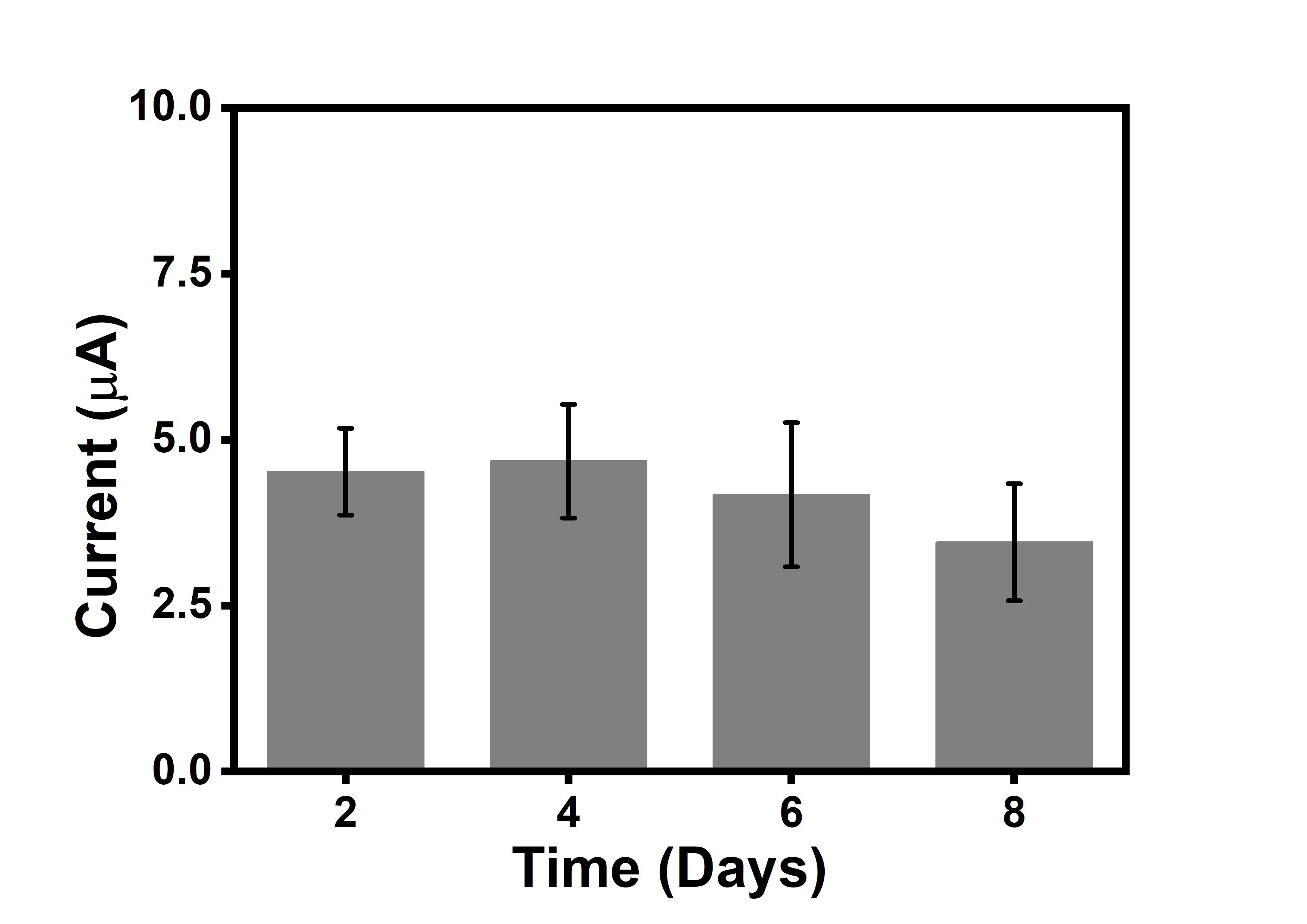


Figure S6. The stability of the proposed LDT-CHA platform, error bars represent standard deviations of the measurements (n = 3).


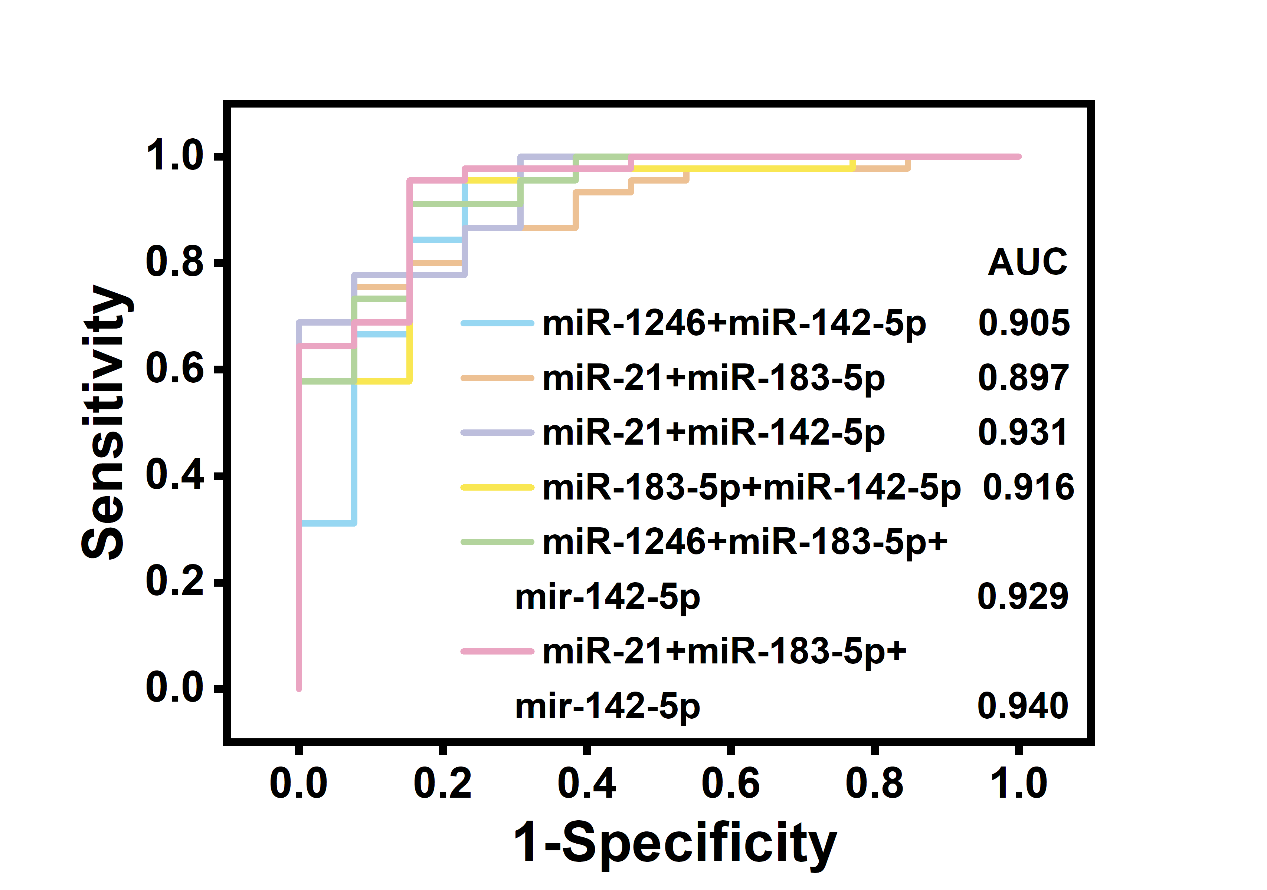


Figure S7. ROC curve of combined sEV-miRNAs for identifying patients with gastric tumors.


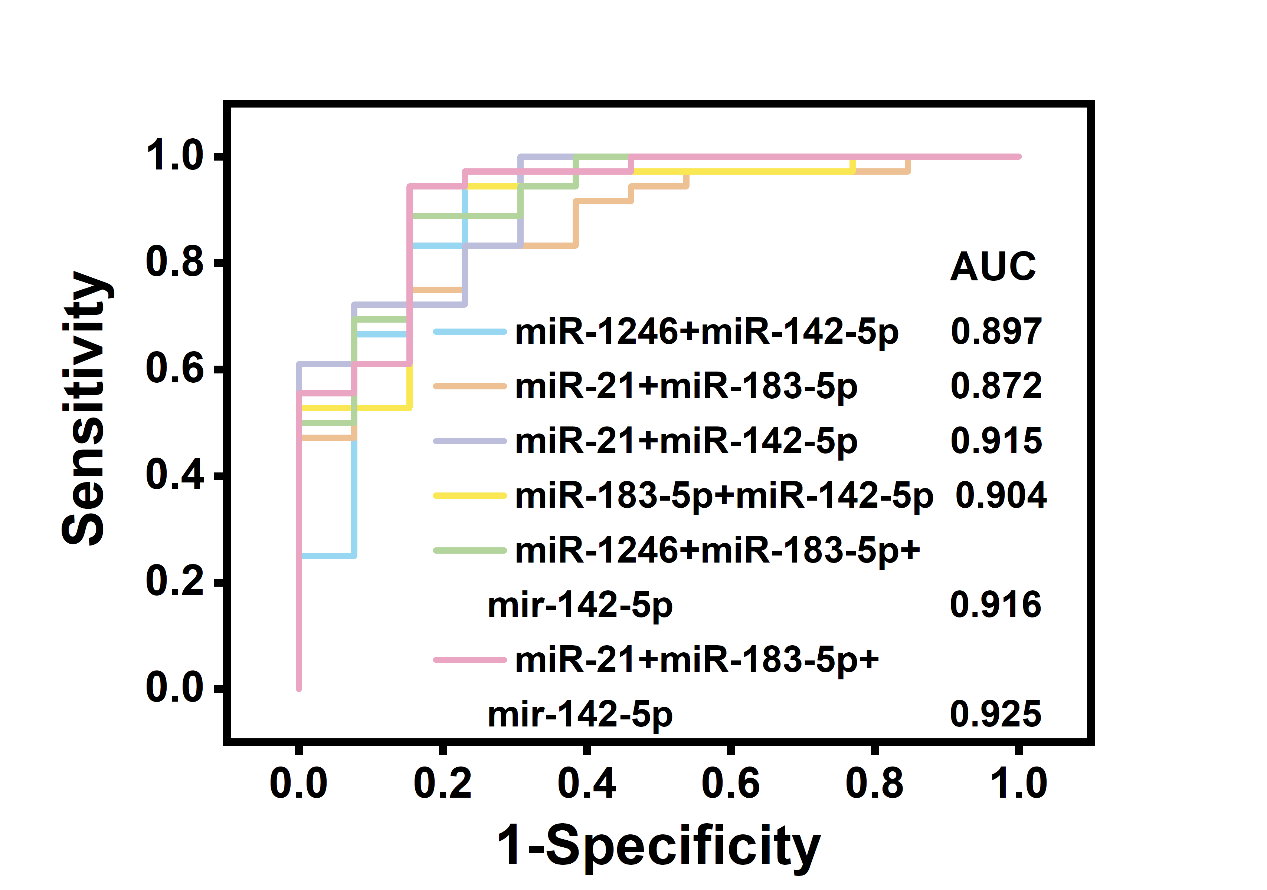


Figure S8. ROC curve of combined sEV-miRNAs for identifying patients with early-stage gastric tumors.

**Supporting Tables.**

Table S1. DNA sequences of used in this assay

| Name | Sequence |
| --- | --- |
| **Capture probe-1** | 5'- GTACATCTGAGTAGACTTCAACACGATTT- SH(CH_2_)_6_ -3' |
| **Capture probe-2** | 5'- TGAAGTCTACTCA -3' |
| **PER-Hairpin-10** | 5'- ATCTCTTATTGGGCCTTTTGGCCCAATAAGAGATAATAAGAGATAGCTTATCA -3' |
| **PER-Hairpin-13** | 5'- ATCTCTTATTTTTGGGCCTTTTGGCCCAAAAATAAGAGATAATAAGAGATAGCTTATCA -3' |
| **PER-Hairpin-15** | 5'- ATCTCTTATTTTTTTGGGCCTTTTGGCCCAAAAAAATAAGAGATAATAAGAGATAGCTTATCA -3' |
| **PER-Primer** | 5'- TTCTCTTATT -3' |
| **17**-**S1** | 5'-TATCACCAGGCAGTTGACAGTGTAGCAAGCTGTAATAGATGCGAGGGTCCAATACTTTGTGTAGCAGGAGAGG -3' |
| **17-S2** | 5'-TCAACTGCCTGGTGATAAAACGACACTACGTGGGAATCTACTATGGCGGCTCTTCTTTAATAAGAGATAATAAGA-3' |
| **17-S3** | 5'-TTCAGACTTAGGAATGTGCTTCCCACGTAGTGTCGTTTGTATTGGACCCTCGCATTTAAGGTTTGACGTGTGG-3' |
| **17-S4** | 5'-ACATTCCTAAGTCTGAAACATTACAGCTTGCTACACGAGAAGAGCCGCCATAGTATT-3' |
| **13-S1** | 5'-ACGAGCGAGTTGATGTGATGCAAGCTGAATGCGAGGGTCCTTTTGTGTAGCAGGAGAGG |
| **13-S2** | 5'- TCAACTCGCTCGTAACTACACTGTGCAATACTCTGGTGACC TTTAATAAGAGATAATAAGA-3' |
| **13-S3** | 5'-TCTGACGTAGTGTATGCACAGTGTAGTAAGGACCCTCGCATTTAAGGTTTGACGTGTGG |
| **13-S4** | 5'- ACACTACGTCAGAACAGCTTGCATCACTGGTCACCAGAGTA |
| **26-S1** | 5'-CGCGCACCTGAGACCTTCTAATAGGGTTGCGACAGTCGTTCAACTAGAATGCCCTTGGGCTGTTCCGGGTGTGGCTCGTCGGTTGTGTAGCAGGAGAGG -3' |
| **26-S2** | 5'-GGCCGAGGACTCCTGCTCCGCTGCGGTTGGCGAACTGGTCCCGTCTACTTACCGTTCCGACGAGCCACACCCGGAACAGCCCTTTAATAAGAGATAATAAGA-3' |
| **26-S3** | 5'-GCCGTAATGTGCATGTATCTCCAGGCTTCCGCAGCGGAGCAGGAGTCCTCGGCCTTGGGCATTCTAGTTGAACGACTGTCGCTAAGGTTTGACGTGTGG -3' |
| **26-S4** | GCCTGGAGATACATGCACATTACGGCTTCCCTATTAGAAGGTCTCAGGTGCGCGTTCGGTAAGTAGACGGGACCAGTTCGCC -3' |
| **1246-H1** | 5’-TTTTTGGAGCAGGGATGTACTAGGCCTGCTCCAAAAATCCATTTTTCCACACGTCAAACCTT-3' |
| **1246-H2** | 5’-CTACTCAGATGTACTAGGTTTTGGAGCAGGCCTAGTACATCCCTGCTTTTCCTCTCCTGCTACACA -3' |
| **miR-21-H1** | 5’-ACAGCCCATCGACTGGTGTTGGATGTACTAGGCAACACCAGTCGATCCACACGTCAAACCTT-3' |
| **miR-21-H2** | 5’-GGTGTTGCCTAGTACATCCAACACCAGTCGATGATGTACTAGGCCTCTCCTGCTACACA-3' |
| **miR-183-5P-H1** | 5’-AGTGAATTGTACCAGTGCCATAGATGTACTAGGTATGGCATCGGTACCCACACGTCAAACCTT-3' |
| **miR-183-5P-H2** | 5’-TGCCATACCTAGTACATCTATGGCACTGGTACGATGTACTAGGCCTCTCCTGCTACACA-3' |
| **miR-142-5P-H1** | 5’-AGTAGTGCTTTCTACTTTATGGATGTACTAGGCATAAAGTAGAAAGCCACACGTCAAACCTT-3' |
| **miR-142-5P-H2** | 5’-CTTTATGCCTAGTACATCCATAAAGTAGAAAGGATGTACTAGGCCTCTCCTGCTACACA-3' |
| **miR-183-5P** | 5’-TATGGCACTGGTACAATTCACT-3' |
| **miR-142-5P** | 5’-CATAAAGTAGAAAGCACTACT-3' |
| **miR-21** | 5’-CAACACCAGTCGATGGGCTGT-3' |
| **miR-1246** | 5’-ATGGATTTTTGGAGCAGG-3' |
| **miR-183-5P-SM** | 5’-TATGGCGCTGGTACAATTCACT-3' |
| **miR-142-5P-SM** | 5’-CATAAAGCAGAAAGCACTACT-3' |
| **miR-21-SM** | 5’-CAACACTAGTCGATGGGCTGT-3' |
| **miR-1246-SM** | 5’-AATGGACTTTTGGAGCAGG-3' |
| **miR-183-5P-DM** | 5’-TATGACGCTGGTACAATTCACT-3' |
| **miR-142-5P-DM** | 5’-CATACAGCAGAAAGCACTACT-3' |
| **miR-21-DM** | 5’-CAACGCTAGTCGATGGGCTGT-3' |
| **miR-1246-DM** | 5’-AATGAACTTTTGGAGCAGG-3' |

Table S2: Clinical information for healthy donors (HD) and patients with gastric cancer (GC).

| Index | Age | Stage |
| --- | --- | --- |
| HD1  HD2  HD3  HD4  HD5  HD6  HD7  HD8  HD9  HD10  HD11  HD12  HD13  HD14 | 58  25  17  36  57  24  35  42  32  56  40  25  65  53 | -  -  -  -  -  -  -  -  - |
| Patient 1 | 45 | I |
| Patient 2 | 49 | I |
| Patient 3 | 59 | IV |
| Patient 4 | 74 | IV |
| Patient 5 | 57 | II |
| Patient 6 | 71 | II |
| Patient 7 | 64 | II |
| Patient 8 | 71 | II |
| Patient 9 | 61 | II |
| Patient 10 | 52 | I |
| Patient 11 | 65 | I |
| Patient 12 | 58 | II |
| Patient 13 | 43 | I |
| Patient 14 | 54 | I |
| Patient 15 | 55 | I |
| Patient 16 | 58 | I |
| Patient 17 | 57 | IV |
| Patient 18 | 48 | II |
| Patient 19 | 65 | I |
| Patient 20 | 64 | I |
| Patient 21 | 59 | III |
| Patient 22 | 64 | II |
| Patient 23 | 68 | II |
| Patient 24 |  | II |
| Patient 25 | 56 | I |
| Patient 26 | 62 | IV |
| Patient 27 | 65 | I |
| Patient 28 | 66 | IV |
| Patient 29 | 80 | I |
| Patient 30 | 30 | I |
| Patient 31 | 74 | III |
| Patient 32 | 37 | I |
| Patient 33 | 59 | II |
| Patient 34 | 70 | II |
| Patient 35 | 64 | II |
| Patient 36 | 49 | I |
| Patient 37 | 57 | I |
| Patient 38 | 62 | I |
| Patient 39 | 55 | IV |
| Patient 40 | 68 | I |
| Patient 41 | 69 | II |
| Patient 42 | 63 | I |
| Patient 43 | 59 | II |
| Patient 44 | 51 | III |

Table S3: Clinical information for patients who underwent clinical treatment comparision

| Index | Age | Stage | CEA | CA-199 |
| --- | --- | --- | --- | --- |
| Patient 1 | 59 | IV | 5.77 | 32.56 |
| Patient 2 | 56 | I | 1.81 | 8.59 |
| Patient 3 | 66 | IV | 14.69 | 5.83 |
| Patient 4 | 80 | I | 2.58 | 19.27 |
| Patient 5 | 30 | I | 0.44 | 5.59 |
| Patient 6 | 74 | III | 130 | 0.57 |
| Patient 7 | 37 | I | 0.94 | 2.77 |
| Patient 8 | 59 | II | 0.93 | 128.97 |
| Patient 9 | 70 | II | 159.2 | 4.61 |

Table. S4 Comparison of different biosensors for detecting sEV-miRNA

| Method | Technique | Reaction Time (min) | Detection range | LOD | Selectivity | Ref. |
| --- | --- | --- | --- | --- | --- | --- |
| RCA with CRISPR/Cas9 | Fluorescence | 180 | 1 pM-10 nM | 90 fM | >1.5 | [^2^](#_ENREF_3) |
| PER | Electrochemical | Not given | 1 fM-1 nM | 0.29 fM | >2.5 | [^3^](#_ENREF_4) |
| Bipedal DNA walkers | Electrochemical | 180 | 0.1 fM -0.1 pM | 67 aM | >12 | [^4^](#_ENREF_5) |
| LSDR | Electrochemical | Not given | 10 fM-70 fM | 2.3 fM | >50 | [^5^](#_ENREF_6) |
| HCR with DNAzyme | Fluorescence | 120 | 10 pM-10 nM | 10 pM | Not given | [^6^](#_ENREF_7) |
| HCR with Exo I | Electrochemical | 180 | 0.1 fM -100 nM | 53 aM | >7.5 | [^7^](#_ENREF_8) |
| AuNPs deposited as nanopillar | SERS | Not given | 1 aM to 100 nM | 1 aM | >5 | [^8^](#_ENREF_9) |
| L-DCDR | Electrochemical | 120 | 0.1 fM-1nM | 65 aM | Not given | [^9^](#_ENREF_10) |
| TMSDR | Electrochemical | Not given | 1 fM-1 nM | 2.75 fM | Not given | [^10^](#_ENREF_11) |
| LDTs-CHA | Electrochemical | 30 min | 100 aM-100 pM | 25 aM | >10 | This work |

Table S5: Cost analysis

| **Step** | **Components** | **Amounts per 100 reactions** | **Costs per 100 reactions ($)** |
| --- | --- | --- | --- |
| Preparation of functional electrodes | DNAs | 10 pmol | 1.84 |
|  | Bst polymerse | 5 pmol | 5.35 |
|  | Reaction buffer | 100 mL | < 0.1 |
| sEV-RNAs isolation | Trizol | 200 mL | 21 |
| Electrochemical analysis | RuHex | 0.25 mg | 3.57 |
|  | Reaction buffer | 1000 mL | < 0.1 |
| **Total cost per 100 reacrtions** | | | **31.72** |

(1) Eldh, M.; Lotvall, J.; Malmhall, C.; Ekstrom, K. Importance of RNA isolation methods for analysis of exosomal RNA: evaluation of different methods. *Molecular immunology* **2012**, *50* (4), 278-286. DOI: 10.1016/j.molimm.2012.02.001. Tang, Y. T.; Huang, Y. Y.; Zheng, L.; Qin, S. H.; Xu, X. P.; An, T. X.; Xu, Y.; Wu, Y. S.; Hu, X. M.; Ping, B. H.; et al. Comparison of isolation methods of exosomes and exosomal RNA from cell culture medium and serum. *International journal of molecular medicine* **2017**, *40* (3), 834-844. DOI: 10.3892/ijmm.2017.3080.

(2) Wang, R.; Zhao, X.; Chen, X.; Qiu, X.; Qing, G.; Zhang, H.; Zhang, L.; Hu, X.; He, Z.; Zhong, D.; et al. Rolling Circular Amplification (RCA)-Assisted CRISPR/Cas9 Cleavage (RACE) for Highly Specific Detection of Multiple Extracellular Vesicle MicroRNAs. *Analytical chemistry* **2020**, *92* (2), 2176-2185. DOI: 10.1021/acs.analchem.9b04814.

(3) Li, X.; Li, X.; Li, D.; Zhao, M.; Wu, H.; Shen, B.; Liu, P.; Ding, S. Electrochemical biosensor for ultrasensitive exosomal miRNA analysis by cascade primer exchange reaction and MOF@Pt@MOF nanozyme. *Biosens Bioelectron* **2020**, *168*, 112554. DOI: 10.1016/j.bios.2020.112554.

(4) Zhang, J.; Wang, L. L.; Hou, M. F.; Xia, Y. K.; He, W. H.; Yan, A.; Weng, Y. P.; Zeng, L. P.; Chen, J. H. A ratiometric electrochemical biosensor for the exosomal microRNAs detection based on bipedal DNA walkers propelled by locked nucleic acid modified toehold mediate strand displacement reaction. *Biosensors & bioelectronics* **2018**, *102*, 33-40. DOI: 10.1016/j.bios.2017.10.050.

(5) Luo, L.; Wang, L.; Zeng, L.; Wang, Y.; Weng, Y.; Liao, Y.; Chen, T.; Xia, Y.; Zhang, J.; Chen, J. A ratiometric electrochemical DNA biosensor for detection of exosomal MicroRNA. *Talanta* **2020**, *207*, 120298. DOI: 10.1016/j.talanta.2019.120298.

(6) He, D.; Hai, L.; Wang, H.; Wu, R.; Li, H. W. Enzyme-free quantification of exosomal microRNA by the target-triggered assembly of the polymer DNAzyme nanostructure. *The Analyst* **2018**, *143* (4), 813-816. DOI: 10.1039/c7an01691c From Nlm.

(7) Guo, Q.; Yu, Y.; Zhang, H.; Cai, C.; Shen, Q. Electrochemical Sensing of Exosomal MicroRNA Based on Hybridization Chain Reaction Signal Amplification with Reduced False-Positive Signals. *Analytical chemistry* **2020**, *92* (7), 5302-5310. DOI: 10.1021/acs.analchem.9b05849.

(8) Lee, J. U.; Kim, W. H.; Lee, H. S.; Park, K. H.; Sim, S. J. Quantitative and Specific Detection of Exosomal miRNAs for Accurate Diagnosis of Breast Cancer Using a Surface-Enhanced Raman Scattering Sensor Based on Plasmonic Head-Flocked Gold Nanopillars. *Small* **2019**, *15* (17), e1804968. DOI: 10.1002/smll.201804968.

(9) Liu, P.; Qian, X.; Li, X.; Fan, L.; Li, X.; Cui, D.; Yan, Y. Enzyme-Free Electrochemical Biosensor Based on Localized DNA Cascade Displacement Reaction and Versatile DNA Nanosheets for Ultrasensitive Detection of Exosomal MicroRNA. *ACS applied materials & interfaces* **2020**, *12* (40), 45648-45656. DOI: 10.1021/acsami.0c14621.

(10) Tang, X.; Wang, Y.; Zhou, L.; Zhang, W.; Yang, S.; Yu, L.; Zhao, S.; Chang, K.; Chen, M. Strand displacement-triggered G-quadruplex/rolling circle amplification strategy for the ultra-sensitive electrochemical sensing of exosomal microRNAs. *Mikrochimica acta* **2020**, *187* (3), 172. DOI: 10.1007/s00604-020-4143-9.
